# Weight Gain, Metabolic Syndrome, and Breast Cancer Recurrence: Are Dietary Recommendations Supported by the Data?

**DOI:** 10.1155/2012/506868

**Published:** 2012-09-24

**Authors:** Colin E. Champ, Jeff S. Volek, Joshua Siglin, Lianjin Jin, Nicole L. Simone

**Affiliations:** ^1^Department of Radiation Oncology, Kimmel Cancer Center and Jefferson Medical College, Thomas Jefferson University, Philadelphia, PA 19107, USA; ^2^Department of Kinesiology, University of Connecticut, Storrs, CT 06269, USA

## Abstract

Metabolic syndrome, which can include weight gain and central obesity, elevated serum insulin and glucose, and insulin resistance, has been strongly associated with breast cancer recurrence and worse outcomes after treatment. Epidemiologic and prospective data do not show conclusive evidence as to which dietary factors may be responsible for these results. Current strategies employ low-fat diets which emphasize supplementing calories with increased intake of fruit, grain, and vegetable carbohydrate sources. Although results thus far have been inconclusive, recent randomized trials employing markedly different dietary strategies in noncancer patients may hold the key to reducing multiple risk factors in metabolic syndrome simultaneously which may prove to increase the long-term outcome of breast cancer patients and decrease recurrences. Since weight gain after breast cancer treatment confers a poor prognosis and may increase recurrence rates, large-scale randomized trials are needed to evaluate appropriate dietary interventions for our breast cancer patients.

## 1. Introduction

Recent data has elucidated the connection between obesity, metabolic syndrome, and poor outcomes in breast cancer patients. Metabolic syndrome, also known as insulin insensitivity syndrome, is defined as central obesity in addition to two of the following risk factors: elevated glucose, insulin resistance, elevated triglycerides, reduced high-density lipoproteins (HDLs), and hypertension [1]. Breast cancer patients with metabolic syndrome undergoing chemotherapy were found to have an overall poor response to treatment and those patients who specifically had high blood glucose levels were noted to have increased rates of disease progression [2]. Additionally, obesity-related variables in early stage breast cancer are significantly associated with recurrence [3]. Data from prospective studies have demonstrated an association between obesity and cancer-specific mortality in multiple sites [4], and weight gain in breast cancer patients is associated with worse outcomes [5]. Prospective data from Italy in patients undergoing surgery and chemotherapy for breast cancer has shown an increase in tumor recurrence in patients with high fasting blood glucose level, low high-density lipoprotein levels, hypertriglyceridemia, large waist circumference, and hypertension [6].

Although dietary strategies are not often imparted to patients completing treatment in the survivorship stage of disease, recently, more effort has been made to invoke dietary modification. Most dietary strategies have focused on reducing fat and increasing fruits, vegetables, and grains in an attempt to decrease recurrence risk. 

This paper serves to review the basic pathways in which obesity and metabolic syndrome may function to increase the risk of recurrence in patients treated for breast cancer and will discuss the current controversy in potential dietary strategies to treat both metabolic and cancer-related issues. 

## 2. Obesity, Weight Gain, and Breast Cancer Outcome

At diagnosis, women with breast cancer who are overweight, obese, or have android body fat distribution experience increased risk of local recurrence, cancer-related death, and overall death by up to 540% [7, 8]. Data have revealed that patients weighing over 130 pounds at cancer diagnosis have inferior recurrence-free survival [9]. Other data point to worse pathological response to chemotherapy in overweight and obese patients [10]. 

However, the effect on outcomes is not only limited to excess weight at presentation, as weight gain after diagnosis of breast cancer negatively impacts disease-free survival, local recurrence, and death as well. In a large cohort of patients followed for nearly 7 years, women who gained more than 5.9 kg after diagnosis were 1.5 times more likely to experience disease recurrence and 1.6 times more likely to die of their disease [11]. In the Nurses' Health Study, which prospectively followed over 5,000 women for several decades, weight and weight gain were associated with increased rates of breast cancer recurrence and mortality [12].

Unfortunately, while weight gain after diagnosis portends a poorer prognosis, the majority of women appear to gain weight after breast cancer diagnosis, with studies showing weight gain occurring in 50–96% of patients [13]. Changes in patient metabolism, physical activity, and dietary intake are proposed mechanisms for this weight gain; however, as discussed below, dietary recommendations may be partly to blame. In fact, most commonly, patients gain over 5.9 kg, which is a risk factor for tumor recurrence [13, 14]. Other studies show that premenopausal women gain significantly more weight than postmenopausal women, and up to 20% gain over 10 kg within 60 weeks of treatment [11]. While weight gain in general is associated with several risk factors for breast cancer recurrence, more specifically, worse outcomes are likely linked to disturbed adipose tissue metabolism resulting from (a) failure to accommodate surplus nutrients in adipose tissue that leads to ectopic accumulation of fat in other tissues and (b) qualitative and/or quantitative changes in physiologic processes, including adipokine production. 

### 2.1. Adipokine Secretion by Adipose Tissue

**Figure 1:**
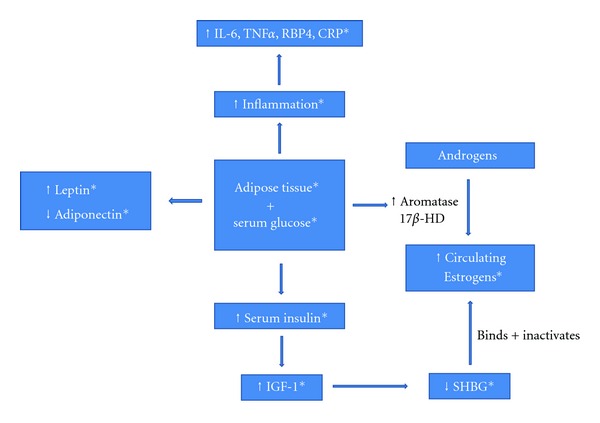
The effect of adipose tissue and serum glucose on several metabolic risk factors for breast cancer recurrence. Legend: the figure above illustrates the interplay between weight gain and several metabolic factors associated with breast cancer recurrence. *represents a factor that is known to influence recurrence. IGF-1: insulin-like growth factor 1 receptor, SHBG: sex hormone-binding globulin, TNF*α*: tumor necrosis factor alpha, IL-6: interleukin 6, RBP4: retinol-binding protein-4, CRP: C-reactive protein, 17*β*-HD: 17*β*-hydroxysteroid dehydrogenase.

 Adipose tissue, especially centrally located adipose tissue, creates several physiological conditions favoring inflammation within the body (Figure 1). Dysregulation of cellular growth, angiogenesis stimulation, and extracellular matrix remodeling favoring tumor progression and recurrence has been shown to result from adipokine secretion from adipose tissue [15]. Fat cells secrete the inflammatory mediators tumor necrosis factor alpha (TNF*α*), interleukin 6 (IL-6), and retinol-binding protein-4 (RBP4). TNF*α* has been shown to promote breast cancer growth through the activation of several intracellular molecular pathways, including MAPK, kappa B (NF-kappa B), and the PI3-K/Akt pathway [16]. Current clinical studies are testing therapeutic TNF*α* inhibitors for metastatic breast cancer, illustrating this relationship [17].

Inflammation is well known to negatively affect the tumor environment, increasing cellular proliferation, tumor survival, and metastasis [18]. Inflammation has also been shown to modify the body's immune response to cancer cells and the response of tumor cells to chemotherapy. Inflammation and proinflammatory cytokines can activate stromal cells in the surrounding extracellular matrix, including fibroblasts, vascular endothelial cells, and macrophages [19]. A growing body of laboratory research has shown that proinflammatory cytokines synthesized by adipose tissue can facilitate tumor growth and metastasis by altering tumor cell biology and activating stromal cells in the tumor microenvironment, such as vascular endothelial cells, tumor-associated macrophages, and fibroblasts.

 Several inflammatory markers have been correlated with worse prognosis in breast cancer patients. Studies in metastatic breast cancer have shown that IL-6 was independently correlated with shorter survival [20, 21]. IL-6 was also found to be predictive of nodal involvement, tumor size at initial diagnosis, progression, and experiencing multiple metastatic sites [22, 23]. Other work has shown C-reactive protein (CRP) to be predictive for survival in newly diagnosed metastatic breast cancer patients [24]. The Glasgow Prognostic Score, which is based on CRP and systemic inflammatory response, significantly predicts for survival in metastatic breast cancer patients [25]. 

In a cohort of over 700 women, circulating levels of CRP and serum amyloid A were associated with reduced disease-free and overall survival. High levels of these inflammatory markers were also associated with a doubling of the risk of recurrence and mortality in a cohort of women diagnosed with breast cancer [26].

Disruption in transcription of IL-6 through gene polymorphisms results in modified serum levels of the cytokine via regulation of gene transcription and has been shown to be associated with disease-free survival [27, 28].

Leptin, another cytokine produced and secreted by adipose tissue, is necessary for normal breast gland development. It is present in normal serum, but can be increased nearly 20-fold in obese individuals [29]. Both in vitro and animal studies have shown that increased levels from excess adipose tissue may activate several molecular and signaling pathways that induce the proliferation of normal and cancer cells, resulting in tumor survival and progression [30]. Leptin has also been shown to stimulate angiogenesis and activate insulin-like growth factor 1 (IFG-1), resulting in increased tumor invasion and metastasis [31].

 Conversely, adiponectin, which is also secreted by adipose tissue, is decreased in obese individuals and those that are insulin resistant. It appears to function in opposition of leptin, blocking its tumorigenic effects, and an increased ratio of leptin to adiponectin leads to tumor proliferation in breast cancer cells [32]. A case-control study found that serum adiponectin is inversely related to breast cancer risk [33].

Overall, strong evidence exists for inflammation leading to poorer outcomes in breast cancer patients and increased rates of recurrence. Excess adipose tissue, most notably in a centralized location, appears to modulate this inflammation, leaving central fat reduction as a potential method on reducing breast cancer recurrence. 

### 2.2. The Effects of Adipose Tissue on Estrogen and Sex Hormones

More than 80% of breast cancers in women older than 45 years of age express receptors including estrogen and other sex hormone receptors [34]. Accordingly, high levels of circulating estrogens have been correlated with an increased risk of breast cancer recurrence after treatment [35]. Increased recurrence is likely a factor of estrogen's effect on cell proliferation through direct stimulation of breast tumor cells and mitogenic activity, as well as through the initiation of increased production of hormones and growth factors that stimulate tumor growth and progression [36]. 

Adipose tissue is capable of peripheral conversion of androgen precursors formed in the adrenal glands and ovaries to estrogen via the enzymes aromatase and 17*β*-hydroxysteroid dehydrogenase [36], serving as an extragonadal source of estrogen production [37]. Obese patients, and notably obese postmenopausal women, have increased levels of the circulating estrogens, estrone, and estradiol and decreased levels of sex hormone-binding globulin (SHBG) [38], which binds and inhibits estradiol. This combination leaves obese patients with elevated active serum estrogens that are potentially capable of fueling tumor progression and recurrence (Figure 1). Lower levels of SHBG also result in increased levels of circulating unbound androgens which may result in tumor progression by themselves and further conversion to estrogens by adipose tissue and associated tumor effects [39]. Furthermore, estrogens upregulate leptin expression, leading to progression of breast cancer cells. Excess adipose tissue has also been shown to result in increased circulating insulin and IFG-1 [40, 41] which results in poorer outcomes and is discussed below. 

### 2.3. Insulin, Insulin Resistance, Blood Glucose, and Breast Cancer Outcome

Biologically, obesity is associated with high levels of circulating insulin, decreased insulin sensitivity, and insulin resistance [42]. Elevated insulin levels with corresponding insulin resistance and elevated blood glucose have been correlated with poor outcomes in breast cancer patients [43–46]. Reasons for poor prognosis have yet to be elucidated and may prove to be multifactorial. Tumor data have revealed that cancer cells exhibit increased glucose consumption [47], and elevated blood glucose may result in the fueling of cancer cells. The Women's Healthy Eating and Living (WHEL) study assessed hemoglobin A1C (HbA1C) in 3,003 survivors with early stage breast cancer and found that elevated serum glucose decreased disease-free survival and significantly reduced overall survival [43]. Though it was not powered to detect a difference in recurrence, the results remain meaningful, revealing a 30% reduction in recurrence rate. Unfortunately, glucose intolerance frequently occurs during adjuvant treatment of breast cancer with chemotherapy [48]. 

As the principle source of energy of cancer cells, elevated serum glucose in itself may fuel tumor progression [49]. Moreover, chronically elevated levels of serum glucose predispose patients to insulin resistance and elevated serum insulin [41], which may exert their own effects on tumor progression. RBP4, the inflammatory mediator mentioned previously, induces insulin resistance, further connecting obesity, inflammation, and insulin resistance. Both insulin and IFG-1 have been shown to increase cell proliferation, protect cells from apoptosis, and induce cellular pathways known to result in worse cancer-related outcomes [46, 50]. As breast tumors often express higher levels of the IGF-1 receptor [51–54], it is of no surprise that insulin and IFG-1 have been correlated with early recurrence and decreased relapse-free survival in breast cancer as well as increased resistance of tumor cells to both chemotherapy and radiation therapy [45, 46]. Elevated levels of insulin also result in the inhibition of SHBG [55] synthesis and therefore an increase in circulating steroids, as described previously. 

In a cohort of over 500 women without known diabetes followed prospectively, fasting insulin levels correlated with distant recurrence and death after breast cancer treatment [44]. Women in the highest quartile of insulin had a twofold increase in the risk of tumor recurrence and a threefold increase in risk of death versus those patients in the lowest quartile. Obese patients also have higher levels of circulating free IGF-1 and serum insulin [40], and a reduction of obesity remains a potential target of decreasing insulin, IGF-1, and insulin resistance. However, it is unclear if such efforts would reduce the risk of recurrence from circulating serum glucose levels and whether this would need to be accomplished through dietary changes.

Recent literature has revealed that diabetic patients on metformin have a lower incidence of invasive breast cancer [56], and those with breast cancer experience higher rates of complete response to chemotherapy [57]. Metformin, which works by sensitizing patients to insulin to lower circulating glucose and insulin levels, further connects metabolic syndrome with treatment outcomes in breast cancer. The effect of insulin on recurrence and survival is being addressed in the recent NCIC Clinical Trials Group MA.32, which is a phase III randomized trial of metformin versus placebo in early stage breast cancer patients. However, it remains unknown if the usage of metformin may benefit patients by mitigating all mechanisms of metabolic syndrome, or if it merely improves insulin resistance. 

## 3. Current Controversies in Metabolic and Weight Management during Breast Cancer

### 3.1. Fear of Weight Loss during Cancer Treatment

Weight loss during cancer treatment was associated with worse outcomes in several cancer sites, as it was a powerful predictor of outcome in lung cancer [58] and was found to be the biological result of increased energy expenditure and malnutrition [59]. Thus attempts were made to limit malnutrition through liberalizing dietary constraints to maximize caloric intake by all potential means, often including high-carbohydrate sources. However, attempts at nutritional intervention to avoid weight loss have thus far provided limited patient benefit [60], as it appears that weight loss and cachexia may be less a function of patient malnutrition and more likely a component of several factors, including the result of aggressive tumor biology and metabolism [61]. In cancers that impair food absorption and the ability to eat, and those with treatment toxicity that limits oral intake, such as esophageal and head and neck cancer, this strategy may be efficacious. However, treatment-related malnutrition is rare in breast cancer patients, with the majority of patients gaining weight during treatment [14], making such a strategy imprudent in breast cancer. 

Accordingly, weight loss may be the result of the aggressive biology and associated decreased survival rather than the cause of the poorer outcomes. Aggressive attempts at weight maintenance and even weight gain during treatment may not serve to help the patient's prognosis, but rather to supply a metabolically active tumor with an increased source of nutrients and fuel. However, an analysis from our institution reveals that online recommendations for cancer patients during treatment and after, including breast cancer patients, often recommend a calorie-dense diet with the purpose of weight maintenance or even weight gain (manuscript currently under review). As a result, women may be following recommendations for patients with cancer of other disease sites, putting them at increased risk of weight gain. 

Moreover, it appears that a majority of women are interested in dietary changes during cancer treatment [62, 63]. Data from our institution reveals that a majority are even interested in engaging in significant dietary changes within the setting of a clinical trial during cancer treatment and after (manuscript currently under review).

### 3.2. Methods of Dietary Intervention

 The abundance of data demonstrating a correlation between both weight gain and metabolic syndrome and inferior treatment outcomes in breast cancer patients has prompted increased evaluation of dietary methods to address these factors. However, controversy exists in defining the appropriate dietary measures to achieve these results.

 Epidemiologic and prospective data have evaluated the relationship between dietary intake, breast cancer recurrence, and overall survival. However, results have thus far been inconsistent [8], likely due to varied study methods. These studies rely on patient self-reporting and food diaries, which are both techniques fraught with error, bias, and inaccuracy [64–67], with the strongest bias towards patient recall of high-fat foods [8]. Epidemiologic data have shown a potential link between dietary fat and breast cancer risk, which has led dietary fat to come under the most scrutiny by prospective studies. Results have been inconsistent with several showing worse outcomes with fat intake. Also, when energy intake is adjusted for, the effect of fat on outcome is no longer significant in many of these studies [8]. 

Epidemiological findings that fruit and vegetable consumption may increase survival after diagnosis of breast cancer [68] have also led to several intervention trials that promote the reduction of fat and its replacement with carbohydrate sources. Interestingly, data show that after breast cancer diagnosis, patients actually appear to make lifestyle changes akin to these recommendations, including increasing fruit and fiber intake and decreasing fat intake [62]. So far, results of these trials have produced no definitive conclusions, with the WHEL study yielding negative results and the Women's Intervention Nutrition Study (WINS) showing that a low-fat diet may decrease breast cancer recurrence [69, 70]. However, weight loss in the WINS study may have been the predominate factor resulting in a reduction in breast cancer recurrence rather than a low-fat diet itself, as patients in the WHEL study, which was negative, gained weight on a low-fat diet [71, 72].

While these trials provide some evidence of guidelines that could be implemented in our patients, from a biological standpoint it seems that there is some cause for concern as this disease derives energy primarily from glucose. Accordingly, several risk factors associated with elevated serum glucose, including elevated insulin and insulin resistance, have been associated with increased recurrence and worse outcomes, as discussed previously. This effect is compounded by the increase in glucose utilization by tumor cells, as tumors derive a majority of their energy from the process of anaerobic metabolism of glucose [49]. Along these lines, tumors need excess amounts of glucose to fuel this inefficient process, and as per data previously, if elevated serum levels are present, these patients may be at increased risk of tumor recurrence. Therefore, replacing dietary fat with carbohydrate sources in cancer patients may be reducing a major source of energy for normal cells and replacing it with fuel for tumor cells. 

### 3.3. What Is the Optimal Dietary Strategy?

**Figure 2:**
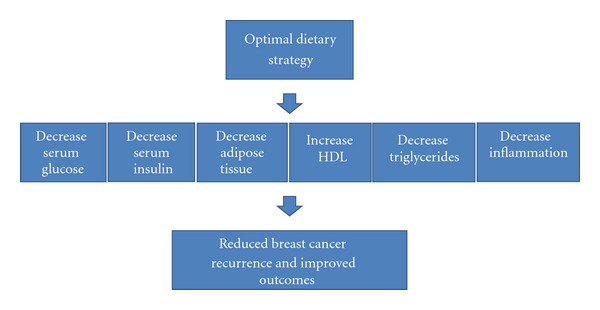
Optimal dietary strategy to reduce breast cancer recurrence risk. Legend: an optimal dietary strategy in reducing breast cancer recurrence would incorporate methods to decrease weight loss, while simultaneously decreasing the risk factors of metabolic syndrome. HDL: high-density lipoprotein, TG: triglycerides.

 A dietary strategy with primary emphasis on decreasing obesity, excess weight, and central adipose tissue would hold the most promise in reducing disease recurrence and improving outcomes, as these factors appear to be strongly correlated with worse outcomes (Figure 2). Decreasing adiposity may result in reduced systemic inflammation, circulating hormones, and insulin. Data from the recent prospective trials described previously, would also favor methods of decreasing serum glucose and insulin levels while increasing insulin sensitivity through diet, all of which are associated with a favorable prognosis. Such factors may actually suggest limiting sugar, carbohydrates, and foods that increase serum insulin and glucose levels. As a result, an optimal diet approach may actually include limiting carbohydrates, and counter to traditional recommendations, replacing these dietary sources with fat and protein. 

### 3.4. Do Recommendations Reflect the Data?

 Currently, physicians typically recommend a low-fat diet with a high consumption of fruits and vegetables, based on epidemiologic and observational data. While a low-carbohydrate, and by default, higher-fat diet is in contradiction to this conventional advice, recent high-level evidence in noncancer patients has shown this diet to produce successful weight reduction in the majority of randomized studies when compared head-to head with a low-fat diet [73]. While several studies have revealed a low-fat and low-carbohydrate diet to be comparable in terms of weight loss, a meta-analysis of 13 randomized trials from 2002 to 2007 revealed superior weight loss with a low-carbohydrate diet versus a low-fat diet at 6 months, though at one year the difference was only found to be 1.05 kg [74]. However, at one year, favorable changes were seen in changes in several risk factors for breast cancer recurrence, including HDL, triglycerides, and systolic blood pressure in the low-carbohydrate groups. Recent trials show more favorable weight reduction with low-carbohydrate dietary strategies [73]. However, while the low-fat diets in these studies were similar to the intervention arms in the WINS and WHEL study, diets in recent randomized trials were followed under close supervision, removing erroneous food questionnaire techniques by providing more accurate methods, including weighed food records and weekly counseling [75, 76]. Yet, not only have these studies illustrated successful weight reduction through a low-carbohydrate diet, but all metabolic factors associated with breast recurrence, including inflammation, central adipose tissue, serum glucose and insulin levels, and insulin resistance were shown to be positively affected through these diets. 

In fact, a recent trial randomized noncancer patients to a low-carbohydrate or low-fat diet with the primary endpoint of improving several metabolic factors that are also risks for breast cancer recurrence. The low-carbohydrate group experienced significantly more weight loss and reduction in adipose tissue, improved blood glucose levels and insulin sensitivity, and increases in HDL, all risk factors for metabolic syndrome and breast cancer recurrence [75]. Also, six markers of inflammation, including TNF and IL-6, underwent a greater decrease in the low-carbohydrate group compared to the low-fat group. These results were confirmed in another randomized trial revealing that a low-carbohydrate diet increased insulin sensitivity by 55%, decreased serum insulin by 50%, while decreasing serum glucose, adiposity, triglycerides, and increasing HDL. Inflammatory markers in this study were evaluated as well, illustrating an improvement in all 16 biomarkers in the low-carbohydrate arm, including RBP4, while the low-fat diet showed no benefit in any of the inflammatory markers [76]. A similar study found that a comparable low-carb diet significantly lowered CRP in patients with high metabolic risk factors [77].

 However, the likely increase in saturated fat that would ensue on a low-carbohydrate diet provides some caution for physicians. Along these lines, the same group compared serum amounts of saturated fat in each diet group [75]. They found that even though the low-carbohydrate group ate three times the amount on saturated fat as the low-fat group, less circulating saturated fat was found in the serum of these patients. It is increased circulating saturated fatty acids, not dietary saturated fat intake, that is associated with increased risk of developing metabolic syndrome [78], diabetes [79], heart attack [80], heart failure [81], and perhaps breast cancer [82]. Of note, the low-fat group consumed a mean 267 g carbohydrates per day while the low-carbohydrate group ate a mean of 45 g. This was likely an effect of decreased levels of serum insulin, as insulin stimulation is known to increase fat storage in adipose tissue, while low insulin levels result in fat oxidation [41]. 

Several other randomized trials have confirmed the superiority of a low-carbohydrate diet in regard to weight loss, decreased serum glucose and insulin levels, increased insulin sensitivity, and decreased blood pressure [83–88]. However, several studies found that when calories were held constant, low-carbohydrate diets were also superior to low-fat diets in regards to weight loss [76, 87, 88], which may point to a metabolic effect connecting insulin, blood glucose, insulin sensitivity, and lipogenesis, all factors that, if reduced, may favorably effect breast cancer prognosis. 

 In conclusion, traditional dietary recommendations of limiting fat while favoring dietary carbohydrates may be a suboptimal strategy for improving nearly every metabolic risk factor for breast cancer recurrence, including decreasing adipose tissue, serum glucose, serum insulin, and inflammatory factors, while increasing HDL and insulin sensitivity. 

## 4. Conclusions

Metabolic syndrome, including weight gain, elevated glucose, and insulin resistance, has been strongly associated with breast cancer recurrence and worse outcomes after treatment. While patients appear to limit fat consumption and increase vegetable and fruit consumption once cancer is diagnosed [62], the majority of patients still gain weight after breast cancer diagnosis. Thus far, dietary intervention trials have been limited, and methods of decreasing metabolic syndrome and obesity in noncancer patients have shown results counter to current dietary strategies in breast cancer patients. Large-scale randomized trials evaluating appropriate dietary interventions to reduce breast cancer recurrence, including a low-carbohydrate approach, are desperately needed. Incorporation of evidence from current randomized trials in noncancer patients may be efficacious.
